# The Replacement Rate: An Imperfect Indicator of Pension Adequacy in Cross-Country Analyses

**DOI:** 10.1007/s11205-015-0892-y

**Published:** 2015-02-10

**Authors:** Filip Chybalski, Edyta Marcinkiewicz

**Affiliations:** Department of Management, Lodz University of Technology, Piotrkowska 266, 90-924 Lodz, Poland

**Keywords:** Pension, Retirement, Consumption smoothing, Replacement rate, Income

## Abstract

Pension systems are usually evaluated from the perspective of two basic criteria: pension adequacy and financial sustainability. The first criterion concerns the level of pension benefits and protection of the elderly from poverty. The second criterion applies to financial liquidity. This paper is primarily of methodological nature. We discuss the problem of measuring pension adequacy, focusing mainly on the replacement rate, which, defined in a number of ways, is the most common measure of pension adequacy. However, as we argue in this paper, it covers only one of its dimensions, namely consumption smoothing. Meanwhile, an equally important dimension, often discussed in the literature and included in most definitions of pension adequacy, is protection of old-age pensioners from poverty. Accordingly, we have proved the thesis that the replacement rate is not a sufficient measure of broadly understood pension adequacy in cross-country studies. Consequently, we have proposed alternative (or possibly complementary) measures called the synthetic pension adequacy indicators (SPAI1-3), defined in basic form as a quotient of relative median income and the at-risk-of-poverty rate. These indicators provide for both the above-mentioned dimensions of adequacy and, according to statistical analysis, also represent them very well. Moreover, the indicators, calculated separately for men and for women, enables evaluation of the third dimension of pension adequacy, namely gender-related differences in pension adequacy.

## Introduction

The adequacy of pension systems is, alongside financial stability and modernisation, one of the targets of pension systems defined by the open method of coordination (OMC).^1^ Adequacy is typically associated with the generosity of pension systems, however, nowadays its evaluation may not be reduced only to the level of pension benefits. The question is whether such evaluation should focus on the income adequacy or rather on the consumption adequacy of a pension system, or perhaps on both. One cannot ignore the phenomenon of the “satisfied poor” or “satisfaction paradox”, which explains high satisfaction among the elderly appearing even at low level of income. On the other hand, higher financial satisfaction in old age can be a result of accumulated wealth (see Hansen et al. 2008). Another important question is: should the adequacy of a pension system be regarded as one-dimensional or multi-dimensional? And, last but not least, how should it be measured? Is the replacement rate, as the most common indicator of the generosity of pension systems, a sufficient measure?

When analysing pension adequacy, two approaches that correspond to its different dimensions can be distinguished. The first one usually involves pension income, which is supposed to reflect the level of income allocation over the life cycle. The measure of pension adequacy applied in this approach is the replacement rate. The second approach, apart from income, also provides for at least one more dimension, namely poverty among pensioners. Sometimes, the income approach is replaced or supplemented by the consumption approach, where the additional measure of pension adequacy is the level of spending among pensioners or in their households. A review of empirical research in the field of pension adequacy leads to the conclusion that the majority of analyses are one-dimensional, and they involve only one measure of pension adequacy which is the replacement rate, although defined in a number of ways. In the most general sense, the replacement rate can be described as the percentage of a pre-retirement income that is paid out as a pension benefit. For example, Biggs and Springstead (2008) analyse pension adequacy solely on the basis of the replacement rate; however, they apply four different measures of income in the pre-retirement period in the denominator of replacement rate. The results of their analysis prove that replacement rates can vary significantly depending on the construction of the indicator. Likewise, Holzmann and Guven (2009) investigate the replacement rate understood as “a useful yardstick for measuring the adequacy of pension benefits, because they express benefits relative to pre-retirement earnings, thereby indicating the degree to which income is replaced when workers retire”. They suggest two main variants of the replacement rate: gross and net, and also involve benefit indexation in their analyses. The Mandatory Provident Fund Schemes Authority (2010) also refers to the major diversification of replacement rates. The paper discusses two approaches to analysing pension adequacy: one based on targeted replacement rates and the other on budgeting. The targeted replacement rate, unlike classic replacement rates, takes into account mainly the material needs of pensioners as the basis for determination of the targeted replacement rate, which is defined as the replacement rate that is required in retirement in order to maintain the same standard of living as during a person’s working years. However, this indicator is hardly a good measure of pension system adequacy in cross-country analyses, as it measures the desirable rather than the actual income of pensioner population. The budgeting approach takes into account the expenses which pensioners are expected to spend in their retirement period. The budgeting methodology employs development of a budget standard, which generally means “a mixture of normative judgments (regarding needs) and empirical data (indicating preferences)” (The Mandatory Provident Fund Schemes Authority  2010). The budgeting approach is also applied by Mutchler et al. (2014), who proposed the Elder Economic Security Standard Index, which benchmarks basic costs of living for elder households. However, it is of very little usefulness in cross-country analyses, as it is incomparable with similar indicators developed for other countries. Furthermore, it is based on survey data, and, as pointed out by Bialowolski and Weziak-Bialowolska (2014), a substantive discrepancy between objective and subjective indicators may occur.

An income and consumption based approach to analysing pension adequacy is also applied by Cole and Liebenberg (2008). They associate pensioner income level with the level of pensioner consumption using two indicators: the income replacement rate and the consumption replacement rate. Hurd and Rohwedder (2008) suggest that consumption adequacy is more important than income adequacy. In their study they define and estimate the measures of economic preparation for retirement based on wealth, which they compared with optimal consumption paths. Studies of this type, involving detailed analyses accompanied by a large number of assumptions, are usually conducted on the national rather than the international level. They are associated with pension planning and decision-making in households and often serve the purpose of increasing social awareness with respect to expected pension earnings. Such an approach to pension adequacy analysis is adopted by VanDerhei (2006) or Bajtelsmit et al. (2013), among others. However, comparative studies in the field of pension adequacy cannot be quite so detailed for several dozen countries, which is why this type of analysis is usually conducted with respect to a single country. This is mainly due to the unavailability of statistical data that meet the adequate comparability criterion required for such analysis.

A comparative pension adequacy analysis based on cross-sectional data for 12 selected countries was conducted by Borella and Fornero (2009). Their study involves comprehensive replacement rates (CORE) based on the comparison (ratio) of living standards after retirement with living standards during the working years. However, they focus only on one dimension of pension adequacy as CORE only applies to the optimal level of consumption smoothing. A number of measures representing the respective dimensions of pension system adequacy in accordance with the objectives of the OMC are included, among others, in the study of the European Commission (2006). However, due to the large number of these indicators, a comparative analysis of pension systems may be complicated. In order to aggregate information on the adequacy contained in these indicators, it is possible, for example, to use relevant multivariate statistical analysis tools. A set of adequacy indicators containing the monitoring indicators used in the OMC and a number of other possible indicators, as well as a method for aggregating them into a synthetic indicator of adequacy of pension system (APS), is proposed by Chybalski (2012). The methodology presented in this study is useful specifically in the case of cross-country analyses. However, due to the fact that the APS measure is relative and based on a synthetic indicator as an aggregate value of the respective adequacy indicators that were subjected to conversion, an increase in the value of the synthetic indicator of APS for one country does not necessarily mean that pension adequacy in that country increased, as this could be the result of reduced adequacy in other countries. Hence, since it is a relative measure, it is sensitive to the variations in the investigated group.

The primary aim of this study is to draw a methodological basis for assessment of pension system adequacy that takes into account the complexity of this concept. Our study contributes to the current literature in two respects. First, we assess the replacement rate as a measure of pension adequacy in cross-country analyses. It is important to distinguish between adequacy evaluation in the case of single-country analyses and in cross-country analyses; the latter require the data to meet the particular criteria. We formulate the thesis that the replacement rate is not a sufficient measure of pension adequacy in cross-country studies, and we attempt to prove the thesis to be correct. Second, we propose our own synthetic pension adequacy indicators, and we demonstrate that they are more accurate, as they, in contrast to the replacement rate, capture different dimensions of pension adequacy.

The paper consists of five sections. In the first one, we introduce the reader to various approaches to measuring pension adequacy presented in empirical research. In the second section, we review the definitions of pension adequacy and define adequacy for the purposes of three-dimensional analysis. In the third section, using statistical tools such as correlation coefficient and regression models for panel data to analyse data from the years 2007–2012 for 29 European countries, we prove the replacement ratio to be an imperfect measure of multidimensional pension adequacy. Section four proposes the synthetic pension adequacy indicators (SPAI1-3) and presents a statistical analysis evaluating the indicators as a representation of two dimensions of pension adequacy. The article ends with synthetic conclusions.

## How To Define Pension Adequacy?

In order to assess whether the replacement rate is a comprehensive and sufficient measure of pension adequacy we must define what pension adequacy is. Since pension adequacy mainly applies to the broader concept of a pension system considered in the micro scale, first we will characterize a pension system as viewed from two perspectives. In the macro scale, it serves as a tool for dividing the current GDP between the working generation and the generation of pensioners (Góra 2008). Such an approach is also represented by Barr and Diamond (2006), who state that future GDP is crucial in pension system analysis, since the consumption of future pensioners will be generated by what is produced in the future, mainly by the generation of their children. From the point of view of an individual person, i.e. in the micro scale, the pension system is a tool of income allocation over the life cycle, a tool of consumption smoothing over the life cycle or a tool of income distribution over time—from the period of economic activity to the period of old age (see Barr 1987; Barr and Diamond 2006; Blake 2006). The theoretical foundations for defining the pension system in the micro scale are thus derived from the theory of consumption and saving, including the life cycle model (LCM) proposed by Modigliani and Brumberg (1954), as well as Ando and Modigliani (1963), and Friedman’s permanent income hypothesis (Friedman 1957). The common feature of both hypotheses is consumption smoothing by an individual based on his or her expectations of the future development of certain economic parameters, including income. It should be noted, however, that LCM usually forms indeed the foundations for pension system analyses and pension decision analyses from the perspective of an individual or a household.

The tool for ensuring pension adequacy, no matter how it is defined and measured, is undoubtedly the broadly understood pension income that provides for consumption on an adequate level. The question is then whether pension adequacy should be measured by a pensioner’s income or by his or her consumption level, or perhaps by both. The difference between income and consumption, both in the period of economic activity and during the retirement period, consists in money saving. While the purpose of money saving is obvious during economic activity, it is to accumulate the pension wealth to be consumed during the retirement period, saving is no longer essential during the retirement period, unless, of course, an individual wishes to save money in order to leave an adequately large bequest. This is consistent with the life cycle model, where an individual, assuming that there is no uncertainty as to the length of life, uses his or her savings in such a way as to fully consume them. An alternative to the assumption of a fixed lifespan is a lifelong annuity that eliminates the risk of too short or too long a lifespan and the impact of the length of life on the level of a pensioner’s income. This way, an individual may smooth his or her consumption throughout his or her entire lifetime.

However, it should be noted that consumption smoothing, which is one of the dimensions of pension adequacy, does not automatically eliminate the risk of poverty, which is its other important dimension. In the case of a system based on the pure defined contribution model, where the pension is a derivative of savings, an individual who generated low income during his or her period of economic activity will still generate low income during the retirement period, even despite proper smoothing of his or her consumption. This is an important argument in favour of using a multi-dimensional approach in pension adequacy analysis that evaluates poverty risk alongside consumption smoothing. The two dimensions of pension adequacy seem indisputable, as is proven by numerous definitions of pension adequacy used by other authors. The World Bank defines an adequate pension system as one that guarantees people, despite the level or form of their economic activity, such a level of benefits as to protect them from poverty and at the same time enable a vast majority of the society to smooth consumption over the life cycle (Holzman and Hinz 2005). The European Commission (2003) defines an adequate pension system as one that “prevents poverty among the elderly; allows people to maintain, to a reasonable degree, their living standard after retirement; and promotes solidarity within and between generations”. The International Labour Organization (ILO) claims that the goal of a pensions system is to guarantee an adequate living standard above the poverty threshold for the rest of a pensioner’s life. Moreover, a pension system should be general and non-discriminatory between genders (Grech 2013). The Organization for Economic Cooperation and Development (OECD) considers an adequate pension system to be one that “replaces a worker’s earnings at a level which enables him or her to maintain a standard of living in retirement comparable to that enjoyed in working life—even though retirement income often do not just replace earnings” (OECD 2013).

The above definitions of pension adequacy formulated by the four large international institutions that collect statistical data regarding the area of pension systems provide foundations for characterizing adequacy. These definitions are quite consistent with respect to two dimensions of pension adequacy: poverty prevention and consumption smoothing over the life cycle. The European Commission also identifies another dimension of pension adequacy: inter- and intra-generational solidarity. However, inter- and intra-generational solidarity could be also perceived as a tool for achieving the goal, which is an adequate pension system, and not as a separate dimension of the adequacy of a pension system. This solidarity is embodied by inter- and intra-generational redistribution. An important feature of pension system adequacy, included in the definition formulated by the OECD, is the fact that income is not the only measure of adequacy and even if it is lower during retirement than during the period of economic activity, it does not necessarily mean that consumption was not adequately smoothed. This is because the measure of consumption is not income as such, but income minus savings, and the demand for money saving during retirement is lower than during the period of economic activity. According to the LCM, savings are only consumed during retirement and their aggregated value is gradually reduced, theoretically, to the zero value at the moment of death. The ILO definition additionally provides for the gender-related dimension of adequacy, stating that one of its targets is to prevent discrimination of women or men in terms of pension benefits. It should be noted that the European Commission does not include, when considering the goals of a pension system related to its adequacy, gender-related discrimination. However, it places this issue in the third group of OMC objectives—modernisation of pension systems (European Commission 2003). Yet the objective of reducing the income gap between men and women during the retirement period concerns adequacy rather than modernisation. Synthesising the above definitions, pension system adequacy should be perceived as a multidimensional category consisting of the following three dimensions:

- Dimension I: Protecting against poverty
- Dimension II: Consumption smoothing
- Dimension III: Differences in adequacy between the genders.

The first and second dimensions are the most important for adequacy evaluation, whereas the third dimension is secondary to them, as it concerns disproportions between men and women in the context of the first two dimensions: poverty and consumption smoothing. Viewed as such, pension adequacy indeed covers the first two dimensions, not only with respect to the total population of pensioners but also separately for men and women. It is only by comparing adequacy in terms of gender that we evaluate the third dimension.

The first dimension—protecting against poverty—characterises the extent to which a pension system protects the elderly against poverty. This dimension does not relate in any way to the period of economic activity or the income generated during that period, as, according to the definitions of adequacy, a pension system should ensure such income in old age as would protect an individual (household) against poverty regardless of his or her professional or income history. Evidently, the objective of a pension system is full protection of the older generation against poverty, which, however, is hardly possible in practice.

The second dimension—consumption smoothing—concerns the period of economic activity, which is a point of reference for evaluation of the level of income or consumption during the retirement period. However, a serious problem appears here. It is difficult to base international analyses on comparable data concerning the consumption of persons of retirement age. Thus, in order to estimate consumption, one should apply income and consumption smoothing level in the life cycle measured by the variability of income throughout the life cycle. This is of course a simplification, but it is necessary in cross-country analyses. Moreover, in many such analyses it is important to group the countries in terms of the analysed characteristic or to analyse the correlations between phenomena based on panel data, rather than to determine or precisely evaluate the level of the analysed variable. Similar is the case with the consumption smoothing level in the life cycle. It should also be noted that the optimum values of income or consumption levels are difficult to identify. The above-mentioned institutions tried, for example, to determine the minimum or optimum level of the replacement rate in a pension system, but the outcomes were inconsistent. The respective institutions proposed different figures as desirable values of the replacement rates. Considering the fact that defining these values is problematic, comparative analyses should assume that higher levels of consumption and income are desired, at least in terms of pension system adequacy, which is the same evaluation perspective as that which is commonly used by pensioners. Thus, we disregard here the effects a pension system, as a tool for dividing the current GDP between generations by payment of pension benefits, has on the economy and labour market, and consequently on the prospects of younger generations.

## Some Empirical Evidence

### The Thesis

Based on a review of literature in the field of pension adequacy evaluation, focusing in particular on the replacement rate as the dominant indicator in this area, we formulate the thesis that the replacement rate is not a sufficient pension adequacy measure in cross-country studies, as it does not capture the complexity of the measured phenomena. This complexity is reflected in the various dimensions of pension adequacy. In the empirical part of our study, we will focus primarily on the first two dimensions, namely protecting against poverty (dimension I) and consumption smoothing (dimension II), treating disproportions in pension adequacy between men and women (dimension III) only as a complementary, rather than the main dimension. The vital character of the first two dimensions is not only due to technical reasons (evaluation of dimensions I and II enables evaluation of dimension III), but also due to substantive reasons. Assessment of poverty among pensioners and pension income in fact renders possible an almost comprehensive evaluation of pension adequacy, while evaluation only of the discrepancies in poverty and income between the genders is practically unreliable because it does not allow for inference as to pension adequacy across the total population.

### Adequacy Indicators Used in the Analysis

In order to prove our thesis, based on Eurostat/EU-SILC^2^ data for the years 2007–2012, we consider a set of statistical indicators that measure pension adequacy. An important selection criterion for these indicators, apart from their information load related to a given dimension of adequacy, is their cross-country comparability represented by the *EU* attribute associated with a given indicator according to standards set by the European Commission (2006). *EU* attribute means that a given indicator is classified among the commonly agreed indicators that allow comparative analysis of the progress made by the respective European countries towards achieving the goals of the OMC. These indicators, unlike those with the *NAT* attribute, enable direct cross-country comparative analyses and have a clear normative interpretation. The criterion of cross-country comparability of the selected criteria is associated with the thesis posed in this paper. We are interested in evaluating the adequacy of a pension system not in one country but in several dozen countries.

In the case of the first dimension of pension adequacy—protecting against poverty—we assume the at-risk-of-poverty rate for pensioners (ARP) to be the most representative indicator. It relates to the group of people whose main activity status is ‘retired’ and it expresses the share of pensioners with an equivalised disposable income below the at-risk-of-poverty threshold. The poverty threshold is assumed to be 60 % of median equivalised income after social transfers. However, it is difficult to state definitely whether this is the actual poverty threshold, as it is a relative value and it is not associated with expenditure but rather with the income of a household. Thus, in one country, income at the level of 60 % of the median income may permit a living standard above the poverty threshold, while in other countries this may not be so. Besides, theoretically, in a country where all live in poverty, according to the methodology adopted by Eurostat, there are always those who earn an income above 60 % of the median income and “theoretically” live outside the poverty zone. Thus, a poverty threshold measured by income (e.g. median income) is imperfect. However, in the Eurostat database there is no better indicator to measure poverty in the pensioner population. Moreover, the at-risk-of-poverty rate for pensioners is an indicator the structure of which allows major independence from the aggregated replacement rate discussed below. According to Eurostat, the cross-country comparability of ARP is high, which means that “data across countries is comparable from 2005 onwards. EU-SILC is based on a common framework defined by harmonised lists of primary and secondary variables, common concepts, a recommended design, common requirements (such as imputation procedures, weighting, sampling error calculation) and classifications aiming at maximising comparability of the information produced”.^3^

Concerning the other dimension of pension adequacy—consumption smoothing—there are two essential measures based on income: the replacement rate (based on income, not on expenditure), which is the most commonly used in studies discussing pension adequacy, as well as the relative median income ratio for the population aged 65+ . As previously mentioned, the replacement rate is defined in a number of ways, by applying different numerators and denominators. However, it is always some kind of relationship between income in the retirement period and income in the period of economic activity. Also, the replacement rate may be computed for the total pensioner population or for its respective cohorts. According to the methodology applied by Eurostat, the aggregated replacement ratio (ARR) is defined as the ratio of the median individual gross pensions of the 65–74 age category relative to median individual gross earnings^4^ of the 50–59 age category, excluding other social benefits. It takes into account gross income, which can be regarded as a disadvantage, because social security contributions paid by pensioners are usually much lower than by working people. Moreover, using gross instead of net income in comparative analyses may be problematic and may distort conclusions relating to countries with a progressive income tax. In addition, the cross-country comparability of this indicator is restricted also by the use of different pension system constructions.^5^ Since the replacement rate is calculated as the median income of persons whose earnings in the retirement period (and in the period of economic activity, too) differ substantially, it does not provide required information on the level of pensioner poverty. Of course, it can be expected that countries with a higher replacement rate have a lower ratio of pensioners living below the poverty threshold, however, this relationship is not at all an evident one. It could be much weaker in a country with strong income asymmetry in the pensioner group. The advantage of such a replacement rate is a broad age range of pensioners and working people whose income (or, more specifically, median income) is provided for in the indicator’s numerator and denominator, respectively. This means that information included in this indicator covers a higher percentage of the surveyed population, but consequently, it is less detailed, e.g. compared to replacement rates calculated separately for respective age cohorts. However, considering the fact that according to Eurostat this rate is meant to be used mainly in comparative cross-country analyses, which refer to the achievement of OMC objectives, such a construction is justified. Currently, it is the only replacement rate calculated for the purpose of monitoring OMC objectives. Another rate, the aggregate replacement ratio (including other social benefits), although it is provided for in the OMC set of indicators, is currently being developed.

Another method to measure pensioner income, alternative or complementary to the replacement rate in evaluating the second dimension of pension adequacy is the relative median income ratio for the population aged 65+ (RMI), which is defined as the ratio of the median equivalised disposable income^6^ of persons aged 60 and over to the median equivalised disposable income of persons aged between 0 and 59. The structure of this indicator is fully consistent with the macro-scale definition of the pension system, and the denominator of this indicator provides for median disposable income of persons aged between 0 and 59. It should be noted that while ARR provides for gross income without any additional social benefits, the RMI provides for total income after tax divided by the number of the members of a household, according to OECD methodology, and as such it may be complementary to the replacement rate. However, the cross-country comparability of RMI is high (not restricted as in the case of ARR). Nevertheless, the complementarity of the two indicators must not be assumed a priori, since their similarities may be evaluated only through a statistical analysis of their data series. Their definitions and calculation methods suggest that rankings of the surveyed countries with respect to the two indicators may be to some extent divergent.

The last dimension of pension system adequacy—income disproportions between the genders—may be measured, according to Eurostat methodology, by absolute differences between the values of relevant indicators described herein above, but calculated separately for men and women. These are:

- Gender differences in the at-risk-of-poverty rate of elderly people 65+ (ARP_GD),^7^
- Gender differences in the aggregate replacement ratio (ARR_GD),
- Gender differences in the relative median income ratio of elderly people 65+ (RMI_GD).

These indicators are used as a consequence of the choice of indicators to measure the first two dimensions of pensions adequacy and their application does not require any further justification. Assessment of differences in the poverty and income levels between men and women is associated with the assumption that pension systems should function in such a way as to ensure the right level of adequacy regardless of gender.

### Data and Methods

Our analysis is focused on cross-country correlations between the above indicators. The research employs a cross-sectional time series (panel data) in the years 2007–2012 drawn from the Eurostat statistical database. The analysis covers 29 European countries: Austria, Belgium, Bulgaria, Czech Rep., Cyprus, Denmark, Estonia, Finland, France, Germany, Greece, Hungary, Iceland, Italy, Latvia, Lithuania, Luxembourg, Malta, Netherlands, Norway, Poland, Portugal, Romania, Slovakia, Slovenia, Spain, Sweden, Switzerland and the United Kingdom.

In order to obtain more robust results, our correlation analysis is based on two independent study procedures. The first one is founded on Spearman’s rank correlation coefficient, which is the nonparametric version of the Pearson correlation coefficient and measures the strength of association between two ranked variables. This coefficient was used to analyse pairs of the above-mentioned indicators in order to verify the similarity of rankings of the surveyed countries with respect to these indicators, disregarding the analytical form of possible correlation. In the case of pairs of indicators that remain in a positive relationship, a Spearman’s rank correlation coefficient close to 1 means a large convergence of the rankings. In the case of negatively correlated pairs, a Spearman’s coefficient close to −1 denotes a large convergence of the rankings. A low absolute value of the Spearman’s rank correlation coefficient suggests lack of convergence between rankings. We calculate the correlation coefficient separately for each year (cross-sectional data) and jointly for all the countries (panel data). The results of the analysis are presented in Table 1. The other procedure aimed at correlation analysis is based on panel regression of the following dependent variables: ARR_total, ARR_male, ARR_female, RMI_total, RMI_male, RMI_female, ARP_total, ARP_male, ARP_female, ARR_GD, RMI_GD, and ATP_GD. In total, we estimate 12 models. For models based on the panel data the test for the variance of the intercept in groups, the Breusch–Pagan test, the Hausman test and the Wald test were applied in order to select the proper form of the model: with fixed effects (FE) or random effects (RE), and with or without time effects (Ajmani 2009). With regard to time effects, initially we assumed that they should be insignificant because intuition suggests that in the period of few years the changes in pension system, if even they occur, should be rather very weak. This results from the fact that pension systems usually evolve smoothly and reforms to them show their effects in the long, not the short term. The Wald test generally confirmed our assumptions, therefore we decided not to include time effects. The results of these estimations are presented in Table 2.

**Table 1:** Spearman’s rank correlation coefficient between variables characterising pension adequacy

Variables	2007	2008	2009	2010	2011	2012	2007–2012
ARR_total and RMI_total	**0.614**	**0.620**	**0.636**	**0.617**	**0.709**	**0.807**	**0.700**
ARR_total and ARP_total	**−0.727**	**−0.727**	**−0.691**	**−0.661**	**−0.611**	**−0.628**	**−0.719**
RMI_total and ARP_total	**−0.747**	**−0.710**	**−0.758**	**−0.719**	**−0.663**	**−0.676**	**−0.729**
ARR_male and RMI_male	**0.807**	**0.668**	**0.799**	**0.719**	**0.826**	**0.834**	**0.776**
ARR_male and ARP_male	**−0.662**	**−0.554**	**−0.665**	**−0.508**	**−0.395**	**−0.426**	**−0.560**
RMI_male and ARP_male	**−0.682**	**−0.636**	**−0.693**	**−0.608**	**−0.602**	**−0.581**	**−0.659**
ARR_female and RMI_female	**0.455**	**0.511**	**0.510**	**0.475**	**0.527**	**0.583**	**0.544**
ARR_female and ARP_female	**−0.612**	**−0.654**	**−0.446**	**−0.556**	**−0.594**	**−0.424**	**−0.585**
RMI_female and ARP_female	**−0.726**	**−0.765**	**−0.767**	**−0.732**	**−0.662**	**−0.690**	**−0.745**
ARR_GD and ARR_total	0.335	0.031	**0.450**	0.201	0.154	0.191	**0.230**
ARR_GD and RMI_total	**0.463**	0.225	**0.546**	0.279	0.299	0.235	**0.336**
ARR_GD and ARP_total	−0.276	0.113	**−0.420**	0.043	0.252	0.103	−0.013
RMI_GD and ARR_total	0.230	0.267	0.251	0.264	0.292	0.321	**0.319**
RMI_GD and RMI_total	**0.442**	0.325	0.318	**0.387**	**0.375**	**0.395**	**0.407**
RMI_GD and ARP_total	−0.160	0.060	0.034	0.042	−0.043	−0.077	−0.077
ARP_GD and ARR_total	0.043	0.105	−0.114	−0.010	0.070	−0.076	0.055
ARP_GD and RMI_total	0.020	0.242	0.072	0.080	0.167	0.010	0.138
ARP_GD and ARP_total	−0.059	−0.352	−0.108	−0.274	−0.430	−0.233	**−0.297**

Correlation coefficients statistically significant at the significance level 0.05 are bolded

**Table 2:** Panel regression models for variables characterising pension adequacy

Dependent variable	Independent variables	Parameter	Standard error	*p* value	FE/RE	R-squared F-statistics *p* value
ARR_total	Const	0.991	6.568	0.880	RE	0.588
	**RMI_total**	**0.601**	**0.067**	**<0.00001**		122.188
	ARP_total	−0.042	0.060	0.485		<0.000
ARR__male	Const	2.849	7.395	0.701	RE	0.624
	**RMI_male**	**0.575**	**0.073**	**<0.00001**		142.037
	ARP_male	−0.077	0.081	0.341		<0.000
ARR_female	Const	11.522	9.655	0.234	RE	0.365
	**RMI_female**	**0.498**	**0.102**	**<0.00001**		49.065
	ARP_female	−0.052	0.081	0.525		<0.000
RMI_total	Const	65.093	3.669	<0.00001	RE	0.701
	**ARR_total**	**0.536**	**0.059**	**<0.00001**		200.226
	**ARP_total**	**−0.492**	**0.042**	**<0.00001**		<0.000
RMI_male	Const	78.512	3.712	<0.00001	FE	0.941
	**ARR_male**	**0.356**	**0.060**	**<0.00001**		76.476
	**ARP_male**	**−0.669**	**0.059**	**<0.00001**		<0.000
RMI_female	Const	79.531	3.048	<0.00001	RE	0.660
	**ARR_female**	**0.242**	**0.048**	**<0.00001**		165.622
	**ARP_female**	**−0.555**	**0.035**	**<0.00001**		<0.000
ARP__total	Const	98.818	4.103	<0.00001	FE	0.928
	ARR_total	−0.006	0.108	0.958		61.205
	**RMI_total**	**−0.968**	**0.085**	**<0.00001**		<0.000
ARP_male	Const	77.580	3.930	<0.00001	RE	0.479
	ARR_male	−0.066	0.067	0.326		78.741
	**RMI_male**	**−0.684**	**0.060**	**<0.00001**		<0.000
ARP_female	Const	109.688	4.845	<0.00001	FE	0.920
	ARR_female	−0.001	0.076	0.991		54.996
	**RMI_female**	**−1.111**	**0.074**	**<0.00001**		<0.000
ARR_GD	Const	−17.175	13.728	0.213	RE	0.082
	ARR_total	−0.264	0.163	0.107		7.616
	**RMI_total**	**0.371**	**0.164**	**0.025**		<0.001
	ARP_total	0.051	0.133	0.701		
RMI_GD	Const	−25.845	20.759	0.215	FE	0.786
	**ARR_total**	**0.852**	**0.243**	**0.001**		16.852
	RMI_total	−0.062	0.264	0.816		<0.000
	ARP_total	−0.266	0.188	0.159		
ARP_GD	Const	−7.586	12.026	0.529	RE	0.084
	ARR_total	−0.079	0.141	0.575		7.807
	RMI_total	0.091	0.147	0.540		<0.001
	**ARP_total**	**−0.291**	**0.112**	**0.010**		

RE denotes random effects and FE denotes fixed effects. Variables and their parameters estimates statistically significant at the significance level 0.05 are bolded

Our analysis does not take into account causality between the variables, but only the correlation between them (in the sense of similarity in their changes). This is because causal dependencies analysis would require involving a number of other variables that, according to the theory and practice, could affect the level of pensioner income and at the same time the aggregated replacement ratio, relative median income and the at-risk-of-poverty rate of pensioners. However, this issue is not relevant to this paper.

### Results

Our analysis was preceded by dispersion coefficients calculations for ARR, RMI and ARP indicators. These reveal discrepancies between the coefficients of variation for the three analysed ratios. The RMI indicator is the least variable as its coefficient of variation fluctuates at the level of 0.11–0.13 and is quite convergent across the surveyed groups (all pensioners, retired men, retired women). The aggregated replacement ratio is slightly more varied (0.14–0.22) across the surveyed groups than in the case of the ARR indicator. The ARP indicator is definitely the most variable in the cross-country analysis, incomparably more than RMI and ARR. Its coefficient of variation oscillates at the level of 0.47–0.70 and is the highest for men. The fact that the indicator representing the first dimension of pension adequacy is much more variable compared to the indicators representing its second dimension means that the surveyed countries differ more in terms of the level of pensioner poverty than the level of pensioner income, which is a measure of the rate of consumption smoothing in the life cycle.

The results presented in Tables 1 and 2 lead to a number of conclusions that support the thesis that the aggregated replacement ratio is not a sufficient or fully representative measure and that it does not fully characterise pension adequacy. Spearman’s correlation coefficients are statistically significant for all analysed pairs of variables representing the first and the second dimension of pension adequacy (ARR, RMI and ARP). However, their values prove only a moderate non-parametric correlation between the ARR and the RMI, and between the ARR and ARP, both for indicators relevant to the total population and for each gender. When comparing the correlation between ARR and ARP (total, male and female) with RMI and ARP (total, male and female) an important conclusion can be drawn. RMI is more correlated with ARP than with ARR. This means that RMI reflects the poverty among pensioners better than ARR, especially with respect to the female group.

The correlation is weak or it does not exist at all between disproportionate indicators of income and poverty between men and women (ARP_GD, ARR_GD, RMI_GD) and income and poverty indicators for the total pensioner population (ARP_total, ARR_total, RMI_total). This confirms that inference on the basis of indicators reflecting gender differences in pension adequacy across the total population is unjustified.

Additionally, an analysis of the results of the panel regression model estimation leads to the conclusion that the aggregated replacement ratio is quite well explained by the other variable representing the same dimension of adequacy, namely the relative median income, but it is not explained, in the same model, by the at-risk-of-poverty rate. RMI also explains well the variable of the at-risk-of-poverty rate of elderly people 65+. Interestingly, the statistically significant variables in the model for the RMI indicator are both ARR and ARP. The above results relate both to the models for the total population and to the models for the respective genders (male and female) and suggest that the relative median income is a variable that quite well reflects both dimensions of pension adequacy—protecting against poverty and consumption smoothing—better than the most commonly used indicator, i.e. the replacement rate. Moreover, the model for ARR_GD suggests that in countries with a higher relative median income (RMI_total) the difference between the aggregated replacement ratio between men and women is greater. Interestingly, ARR_total is a statistically insignificant variable in this particular model. On the other hand, in the model for RMI_GD, ARR_total is statistically significant and RMI_total is not. Thus, in countries with a higher replacement rate in the total pensioner population, the difference in the relative median income between men and women is also greater. Neither ARR_total, nor RMI_total explain the differences in poverty between men and women aged 65+.

The synthetic conclusion that may be drawn from the above statistical and econometric analysis of the main indicators of pension system adequacy, understood as a three-dimensional phenomenon, is somewhat surprising, as it suggests that the aggregated replacement ratio, at least as calculated by Eurostat based on EU SILC data, is not the best measure of pension adequacy, considering two of its dimensions: protecting against poverty and consumption smoothing. This confirms our thesis. A better measure is the relative median income, as it better explains, with respect to the first two dimensions of adequacy, the variables ARR_total and ARP_total, and at the same time is itself explained by the two variables. The first two dimensions of pension adequacy generally do not reflect the third dimension and thus ARR and RMI are not sufficient measures of poverty and income disproportions between genders in the population of pensioners.

## The Proposition of the Simple Synthetic Pension Adequacy Indicator (SPAI)

The method of measuring pension adequacy we propose is intended to meet the following criteria:

- Criterion 1: provide for the first and second dimension of pension adequacy,
- Criterion 2: draw on raw, not standardised data so as to limit the relativity of measurement,
- Criterion 3: draw on data comparable across countries.

The correlation analysis suggests that it is possible to construct a simple indicator that would allow measurement of the first two dimensions of pension adequacy: protecting against poverty and consumption smoothing. On the other hand, indicators relevant to the total pensioner population may not be directly applied to evaluate the third dimension of pension adequacy, namely gender disproportions, as the indicators of these disproportions (between the genders) are not sufficiently correlated to the indicators relevant to the total population. Thus, the first and second dimension may be evaluated jointly, while the third dimension should be evaluated separately.

Of the two income indicators measuring consumption smoothing, namely the aggregated replacement ratio and relative median income, the latter seems to be more informative (mainly due to the fact that it takes into account disposable income after tax and other deductions). Therefore, in our proposition of the simple synthetic pension adequacy indicator (SPAI) information provided by the ARP ratio is supplemented by the RMI ratio.

$$SPAI1 = \frac{RMI}{ARP}$$

This indicator may be calculated both for the total pensioner population and for men and women separately. It is an uninterpretable measure and only serves the purpose of comparing pension adequacy. However, with this indicator, it is possible to simultaneously evaluate the first two dimensions of adequacy as well as its third dimension, when calculated separately and compared for men and women. It has no units assigned, and theoretically ranges between 0 and infinity, but it can be calculated providing that ARP is not equal to zero, which is practically always the case. Higher values of the SPAI indicator mean greater pension adequacy in the surveyed population. Moreover, the proposed indicator calculated for a given country in comparative analyses is resistant to changes in pension adequacy in other analysed countries due to the fact that its components do not require standardisation. Thus, an increase in the indicator in a given country always means an increase in pension adequacy resulting from higher pensioner income (expressed as RMI) or reduction of poverty (expressed as ARP), regardless of changes, if any, in these indicators in the other analysed countries.

An analysis of Spearman’s rank correlations (Table 3) suggests that the proposed SPAI indicator (calculated for the total population and separately for men and women) is highly correlated with all the sub-indicators of pension adequacy. Moreover, changes in it are accompanied at most by the changes in the at-risk-of-poverty rate of elderly people 65+ (ARP), as the Spearman’s rank correlation coefficient between the two variables is nearly equal to −1. Importantly, this indicator, even though its structure does not include the aggregated replacement ratio (ARR), is quite strongly correlated with the latter, meaning that it is a good representative of that ratio. All the signs of this indicator’s correlation coefficients with both sub-indicators are as expected, i.e. they are positive for the pair SPAI and RMI and negative for the pair SPAI and ARP, which is another proof that the indicator has the proper structure. Thus, it can be stated that the proposed indicator is appropriate for measuring the first two dimensions of pension adequacy.

**Table 3:** Spearman’s rank correlation coefficient between SPAI1 indicator and selected sub-indicators of pension adequacy

Variables	2007	2008	2009	2010	2011	2012	2007–2012
SPAI_total and ARR_total	**0.750**	**0.721**	**0.686**	**0.676**	**0.637**	**0.681**	**0.740**
SPAI _total and RMI_total	**0.785**	**0.782**	**0.836**	**0.776**	**0.743**	**0.778**	**0.802**
SPAI _total and ARP_total	**−0.994**	**−0.989**	**−0.982**	**−0.986**	**−0.983**	**−0.979**	**−0.989**
SPAI _male and ARR_male	**0.681**	**0.609**	**0.699**	**0.558**	**0.467**	**0.549**	**0.609**
SPAI _male and RMI_male	**0.713**	**0.712**	**0.723**	**0.661**	**0.660**	**0.646**	**0.704**
SPAI _male and ARP_male	**−0.995**	**−0.987**	**−0.992**	**−0.989**	**−0.989**	**−0.972**	**−0.993**
SPAI _female and ARR_female	**0.619**	**0.650**	**0.457**	**0.554**	**0.597**	**0.513**	**0.601**
SPAI _female and RMI_female	**0.782**	**0.788**	**0.805**	**0.771**	**0.722**	**0.761**	**0.804**
SPAI _female and ARP_female	**−0.992**	**−0.996**	**−0.988**	**−0.990**	**−0.982**	**−0.959**	**−0.991**

Correlation coefficients statistically significant at the significance level 0.05 are bolded

**Fig. 1:**
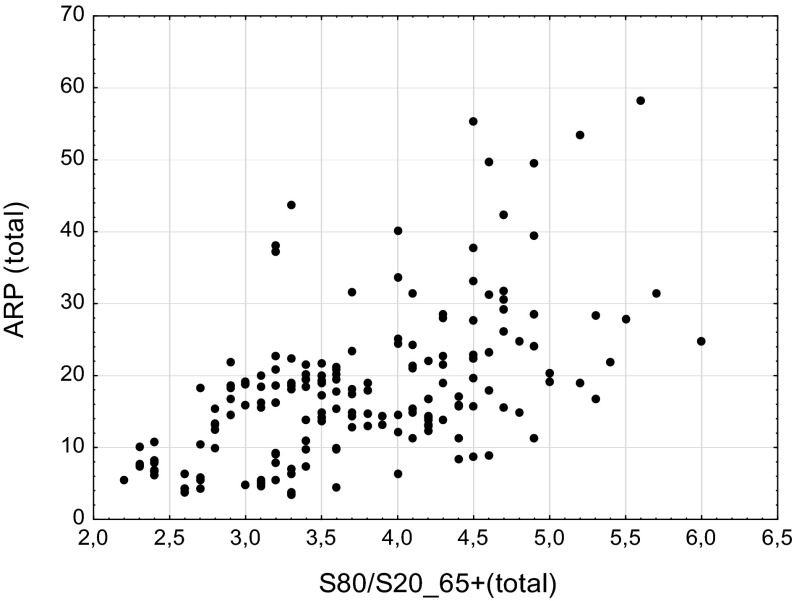
Correlation plot for poverty (ARP_total) and income inequality (S80/S20_65+)

Taking into account the possible sensitivity of the poverty rates to income inequality (see e.g. Immerfol et al. 2006), which is also observed in our data to some extent (Fig. 1), we propose two developments of SPAI1 including inequality of income distribution indicator S80/S20. This is the ratio of total income received by the 20 % of the population with the highest income (top quintile) to that received by the 20 % of the population with the lowest income (lowest quintile) and is of high comparability.^8^ The first one—SPAI2—takes into account only income inequality in the population aged 65+ and has following formula:

$$SPAI2 \,=\, \frac{RMI}{ARP \cdot S80/S20\_65 + }$$

The second one—SPAI3—includes income inequality not only for the elderly, but also for the population aged 65−, which reflects the fact that pension systems are the tool of dividing GDP (national income) between young and old generations. It justifies the evaluation of pension adequacy taking into account also the incomes of younger cohorts since a pension system exists in a given economic environment. This approach is reflected also in RMI and ARR, as they are a ratio of a given income measure in older and younger populations. SPAI3 indicator is expressed by the formula:

$$SPAI3 = \frac{RMI}{ARP} \cdot \frac{S80/S20\_65 - }{S80/S20\_65 + }$$

The concept of SPAI2 and SPAI3 differs in the approach to the evaluation income inequality in the case of pension system analysis. The former indicator refers to the income inequality among the elderly. This inequality is very important in pension adequacy evaluation since ARP, as a measure of relative poverty (the poverty threshold equals 60 % of median income in population), does not supply information on what the income of people living below poverty line is in comparison to the income of people living over it. The highly developed indicator—SPAI3—takes into account income change after retirement rather than income inequality *per se*. Obviously, interpreting the ratio$\frac{S80/S20\_65 - }{S80/S20\_65 + }$ as a change is a simplification, because we assume that the generation of people aged 65 and less would experience the same income inequality as the generation of present pensioners (population aged 65+). In the short term, this seems to be justified, e.g. the cohort of people aged 64 today in the next year will be aged 65 and therefore they will change the group for which S80/S20 is calculated (from 65− to 65+). According to pension system adequacy and its two main dimensions, the relation between income disproportions in the two mentioned generations expresses also the redistribution in a pension system supporting mainly poverty alleviation. The greater the ratio $\frac{S80/S20\_65 - }{S80/S20\_65 + }$, the higher the redistribution reducing income inequality among pensioners (population aged 65+). If this ratio equals unity, which means that income inequality in both 65− and 65+ population is at the same level (inequality does not change after retirement), SPAI1 = SPAI3.

All the proposed synthetic indicators are based on four Eurostat/EU-SILC measures of income and poverty: RMI, ARP, S80/S20_65− and S80/S20_65+ which are geographically comparable since they have *EU* attribute in overarching portfolio. As these indicators are the basis on which the proposed three synthetic pension adequacy indicators are built, all the SPAI indicators seem to hold the criterion 3 and therefore to be comparable in cross-country studies.

As in the case of SPAI1, Spearman’s rank correlation coefficients between SPAI2 and SPAI3, and given adequacy indicators, were calculated (see Tables 4, 5).

**Table 4:** Spearman’s rank correlation coefficient between SPAI2 indicator and selected sub-indicators of pension adequacy

Variables	2007	2008	2009	2010	2011	2012	2007–2012
SPAI2 (total) and ARR (total)	**0.703**	**0.702**	**0.649**	**0.679**	**0.578**	**0.570**	**0.700**
SPAI2 (total) and RMI (total)	**0.696**	**0.646**	**0.703**	**0.700**	**0.628**	**0.616**	**0.676**
SPAI2 (total) and ARP (total)	**−0.975**	**−0.982**	**−0.964**	**−0.976**	**−0.982**	**−0.979**	**−0.981**
SPAI2 (male) and ARR (male)	**0.642**	**0.507**	**0.644**	**0.502**	0.361	**0.414**	**0.545**
SPAI2 (male) and RMI (male)	**0.598**	**0.603**	**0.660**	**0.590**	**0.552**	**0.475**	**0.611**
SPAI2 (male) and ARP (male)	**−0.973**	**−0.987**	**−0.976**	**−0.980**	**−0.988**	**−0.993**	**−0.990**
SPAI2 (female) and ARR (female)	**0.625**	**0.633**	**0.428**	**0.497**	**0.536**	**0.414**	**0.569**
SPAI2 (female) and RMI (female)	**0.683**	**0.691**	**0.655**	**0.667**	**0.564**	**0.560**	**0.676**
SPAI2 (female) and ARP (female)	**−0.976**	**−0.973**	**−0.954**	**−0.972**	**−0.962**	**−0.983**	**−0.976**
SPAI2 (total) and S80/S20_65+ (total)	**−0.763**	**−0.707**	**−0.620**	**−0.665**	**−0.644**	**−0.483**	**−0.670**
SPAI2 (male) and S80/S20_65+ (male)	**−0.831**	**−0.722**	**−0.707**	**−0.796**	**−0.764**	**−0.595**	**−0.732**
SPAI2 (female) and S80/S20_65+ (female)	**−0.721**	**−0.649**	**−0.559**	**−0.559**	**−0.592**	**−0.528**	**−0.617**

Correlation coefficients statistically significant at the significance level 0.05 are bolded

**Table 5:** Spearman’s rank correlation coefficient between SPAI3 indicator and selected sub-indicators of pension adequacy

Variables	2007	2008	2009	2010	2011	2012	2007–2012
SPAI3 (total) and ARR (total)	**0.679**	**0.711**	**0.651**	**0.682**	**0.629**	**0.596**	**0.703**
SPAI3 (total) and RMI (total)	**0.710**	**0.770**	**0.758**	**0.723**	**0.631**	**0.633**	**0.721**
SPAI3 (total) and ARP (total)	**−0.951**	**−0.927**	**−0.920**	**−0.916**	**−0.964**	**−0.930**	**−0.950**
SPAI3 (total) and ARR (male)	**0.746**	**0.551**	**0.712**	**0.549**	**0.385**	**0.389**	**0.577**
SPAI3 (total) and RMI (male)	**0.677**	**0.689**	**0.690**	**0.622**	**0.568**	**0.539**	**0.652**
SPAI3 (total) and ARP (male)	**−0.899**	**−0.850**	**−0.836**	**−0.790**	**−0.830**	**−0.800**	**−0.851**
SPAI3 (total) and ARR (female)	**0.558**	**0.665**	**0.570**	**0.656**	**0.734**	**0.616**	**0.663**
SPAI3 (total) and RMI (female)	**0.681**	**0.834**	**0.765**	**0.750**	**0.626**	**0.648**	**0.739**
SPAI3 (total) and ARP (female)	**−0.883**	**−0.902**	**−0.867**	**−0.870**	**−0.936**	**−0.840**	**−0.905**
SPAI3 (total) and S80/S20_65+ (total)	**−0.610**	**−0.523**	**−0.459**	**−0.484**	**−0.518**	**−0.414**	**−0.521**
SPAI3 (total) and S80/S20_65− (total)	**−0.408**	−0.258	−0.180	−0.001	0.003	−0.022	−0.126
SPAI3 (male) and S80/S20_65+ (male)	**−0.722**	**−0.606**	**−0.640**	**−0.747**	**−0.684**	**−0.475**	**−0.636**
SPAI3 (male) and S80/S20_65− (male)	**−0.399**	**−0.387**	−0.364	−0.275	−0.179	−0.128	**−0.264**
SPAI3 (male) and S80/S20_65+ (female)	**−0.628**	**−0.511**	**−0.427**	**−0.562**	**−0.543**	**−0.501**	**−0.538**
SPAI3 (male) and S80/S20_65− (female)	**−0.449**	**−0.414**	−0.339	−0.314	−0.286	−0.130	**−0.301**

Correlation coefficients statistically significant at the significance level 0.05 are bolded

The analysis of Spearman’s rank correlation between SPAI2 and SPAI3 and ARR, RMI and ARP shows that these indicators are highly correlated, however not as highly as in the case of SPAI1 (see Table 3). SPAI2 is also significantly correlated with S80/S20_65− whereas in the case of SPAI3 this correlation is weaker, but still significant. The correlation is weak or even does not exist between SPAI3 and S80/S20_65−.

All the proposed synthetic indicators of pension system adequacy are significantly correlated with each other (Spearman’s rank correlation greater than 0.9 in each year as well as in the entire period under analysis). However, SPAI3 seems to be the best one since it includes the most information about pension adequacy: poverty alleviation, consumption smoothing, but also income inequality in population aged 65+ compared to income inequality in population aged 65−. While SPAI1 is mostly correlated with two main pension system dimensions, poverty alleviation and consumption smoothing, it does not provide any information on income inequality or change in it after retirement.

## Conclusions

In this paper we have assessed the replacement rate as the measure of multidimensional adequacy of pension systems in cross-country studies. We state that cross-country analyses require addressing many challenges associated with obstacles mainly of a methodological nature. The most important of these is undoubtedly the need to apply internationally comparable statistical data. Thus, when using data from, for example, Eurostat, it is necessary to use measures of quite a large degree of generality. The same applies to replacement rates, which in the case of longitudinal analyses for a given country are often calculated using a completely different methodology than in the case of replacement rates calculated in cross-country analyses. As a result, the first kind of replacement rates contains much more detailed and relevant information, but only with respect to one country. Another limitation is associated with the requirement to apply income-based rather than expense-based measures of consumption, which is supposed to be smoothed by a pension system.

The discussion and statistical analysis presented in our paper proves the thesis that the replacement rate—the most popular measure of pension adequacy—is not, at least with respect to Eurostat data, a sufficient representative of this adequacy understood in two dimensions: protecting against poverty and consumption smoothing. Concerning consumption smoothing, a good complementary or even alternative measure to the replacement rate may be the relative median income, which, in the case of Eurostat data, is based on net income, not on gross income, as is the case with the aggregated replacement ratio, which is its definite advantage. Moreover, the panel regression analysis shows that it is relative median income, rather than the aggregated replacement ratio, that better explains pensioner poverty, which suggests that comparative analyses based on one indicator, should use the relative median income instead of the replacement rate.

We have also proposed other alternatives to the replacement rate in analyses based on one indicator—the synthetic pension adequacy indicator (SPAI). These measures are a quotient of the relative median income and the at-risk-of-poverty rate of pensioners (SPAI1) completed by inequality of income distribution in population 65+ (SPAI2) and additionally by inequality of income distribution in population aged 65− (SPAI3). An important advantage of these indicators is the fact that they provide for two dimensions of pension adequacy, both protecting against poverty and consumption smoothing, better than replacement rate does. They adequately reflect them, as was proven by statistical analysis, and in the case of SPAI2 and SPAI3, they additionally include information on income inequality, which may impact poverty.

## Footnotes

1 The open method of coordination (OMC) was created as part of employment policy and the Luxembourg process. It has been defined as an instrument of the Lisbon strategy and provides a new framework for cooperation between the Member States, whose national policies can thus be directed towards certain common objectives. Under this intergovernmental method, the Member States are evaluated by one another (http://europa.eu/legislation_summaries/glossary/open_method_coordination_en.htm).

2 The European Union Statistics on Income and Living Conditions (EU-SILC) is an instrument aiming at collecting timely and comparable cross-sectional and longitudinal multidimensional microdata on income, poverty, social exclusion and living conditions. This instrument is anchored in the European Statistical System (ESS). The EU-SILC instrument provides two types of data:

- Cross-sectional data pertaining to a given time or a certain time period with variables on income, poverty, social exclusion and other living conditions,
- Longitudinal data pertaining to individual-level changes over time, observed periodically over a 4-year period.

3 See e.g. information on indicator profile provided by Eurostat (http://epp.eurostat.ec.europa.eu/cache/ITY_SDDS/FR/tsdsc250_esmsip.htm).

4 Gross earnings are paid in cash directly to an employee before any deductions for income tax and social security contributions paid by the employee. All bonuses, whether or not regularly paid, are included (13th or 14th month pay, holiday bonuses, profit-sharing, allowances for leave not taken, occasional commissions, etc.).

5 See e.g. information on indicator profile (http://epp.eurostat.ec.europa.eu/cache/ITY_SDDS/en/tsdde310_esmsip.htm).

6 The equivalised disposable income is the total income of a household, after tax and other deductions, that is available for spending or saving, divided by the number of household members converted into equalised adults; household members are equalised or made equivalent by weighting each according to their age, using the so-called modified OECD equivalence scale.

7 Gender differences in the at-risk-of-poverty rate of elderly people 65+ were used due to the fact that Eurostat does not calculate gender differences in the at-risk-of-poverty rate for pensioners, but it can be assumed that they are to a large extent similar as in most European countries the retirement age is at or around 65 years.

8 See e.g. Eurostat Quality Profile: Inequality of income distribution (http://epp.eurostat.ec.europa.eu/portal/page/portal/sdi/files/QP%20Inequality%20of%20income%20distribution.pdf).
